# A novel GBDT-BiLSTM hybrid model on improving day-ahead photovoltaic prediction

**DOI:** 10.1038/s41598-023-42153-7

**Published:** 2023-09-13

**Authors:** Senyao Wang, Jin Ma

**Affiliations:** School of Electrical and Computer Engineering, The University of Sydney, Sydney, NSW 2008 Australia

**Keywords:** Materials science, Electrical and electronic engineering

## Abstract

Despite being a clean and renewable energy source, photovoltaic (PV) power generation faces severe challenges in operation due to its strong intermittency and volatility compared to the traditional fossil fuel power generation. Accurate predictions are therefore crucial for PV’s grid connections and the system security. The existing methods often rely heavily on weather forecasts, the accuracy of which is hard to be guaranteed. This paper proposes a novel GBDT-BiLSTM day-ahead PV forecasting model, which leverages the Teacher Forcing mechanism to combine the strong time-series processing capabilities of BiLSTM with an enhanced GBDT model. Given the uncertainty and volatility inherent in solar energy and weather conditions, the gradient boosting method is employed to update the weak learner, while a decision tree is incorporated to update the strong learner. Additionally, to explore the correlation between photovoltaic power output and historical time-series data, the adaptive gradient descent-based Adam algorithm is utilized to train the bidirectional LSTM model, enhancing the accuracy and stability of mid- to long-term time-series predictions. A prediction experiment, conducting with the real data from a PV power station in Sichuan Province, China, was compared with other methods to verify the model’s effectiveness and robustness.

## Introduction

### Background

In recent years, the global momentum towards renewable energy has been palpable. In a stark testament to this shift, 2022 saw a 13% surge in renewable energy capacity compared to 2021, reaching nearly 340 GW, with solar PV contributing an unprecedented 220 GW. Further emphasizing this momentum, the REmap 2030 report projects the share of renewable energy in the power sector to rise from 18% in 2010 to 44% by 2030^1^. The “California duck curve”^2^, a distinctive illustration of the timing imbalance between peak demand and renewable energy production, stands as a tangible testament to photovoltaics’ growing influence on grid stability. Contrasting with conventional coal-fired plants that consistently output energy, renewables introduce significant fluctuations due to their weather-dependency^3^. As traditional power units recede into the background, the precision of weather forecasting emerges as a crucial component. In this backdrop, PV generation takes center stage, emphasizing the indispensability of accurate predictions. As renewables continue to carve their space in the energy sector, the development of advanced predictive methodologies is undeniably paramount.

### Literature reviewed and motivation

Amid the rapid transition to renewable energy, precise forecasting of PV generation has solidified its place as a central research challenge. Leveraging advancements in computational technology, our ability to predict with increased accuracy is now within grasp. Enhanced by modern technological innovations, the precision of weather monitoring and data acquisition is paramount in the evolving renewable energy landscape.

There are two major types of PV forecasting models, namely, physical models and statistical models. The physical models are based on the illumination radiation and the weather data near the PV power station. This numerical weather prediction (NWP)model is extremely dependent on the ambient environment measurements. Ref.^4^ proposes a day-ahead photovoltaic power forecasting (PPF) and uncertainty analysis method based on WT-CNN-BiLSTM-AM-GMM. Ref.^5^ also uses accurate radiation data to predict the PV power generation. However, these work only considers solar irradiance. To improve the forecasting accuracy and remove the reliance on a single weather factor, Ref.^6^ considers the weather features comprehensively, including wind force, wind direction, temperature, etc. Furthermore, Ref.^7–10^ consider the effect of cloud cover and capture the cloud behavior to reduce the influence of cloud on the solar irradiation. Getting weather data from satellites directly instead of from nearby weather station could provide more comprehensive weather features, but it also increases the computing burden due to the large volumes of data and unclearness in the correlations between different types of weather data and the PV ouput.

Statistical techniques for PV prediction span traditional methods like ARIMA, Bayesian statistics, and Markov chains, which leverage historical PV outputs for data periodicity modeling as illustrated by Ref.^11^, to advanced machine learning methods Ref.^12^. However, the traditional methods’ efficiency diminishes with swift weather-induced power fluctuations. Contrarily, contemporary machine learning models like LSTM, SVM, Bayes, KNN, and XGBOOST are gaining traction, as showcased by studies such as Ref.^13^. For example, while SVM offers error minimization potential, it remains parameter-sensitive Ref.^14^, and Bayes, effective for small datasets, varies based on input data quality^15^. Particularly, LSTM, a deep learning variant, captures spatial–temporal correlations effectively, evident from Ref.^16^. Yet, given the inherent unpredictability of PV outputs due to weather variations, hybrid models like the one proposed in Ref.^17^ are gaining momentum, emphasizing the need for continued research in this domain to enhance forecast accuracy.

Above all, although the PV forecasting is an important research problem and has been widely studied with the above methods, there are still exist following unsolved problems: (1) The physical NWP model relies on an extensive set of meteorological variables for precise forecasting. When dealing with the absence or incompleteness of certain meteorological data, the model’s forecast accuracy can deteriorate, or it may become infeasible to produce a reliable prediction. (2) Due to the non-stationarity of PV power time series, single model prediction is often less accurate than the combined model, and obtain the biased result^18^. (3) Current models typically encounter difficulties in maintaining prediction accuracy when they lack real-time data adjustments. Without the capability to recalibrate based on live data, the performance of many models deteriorates, especially in terms of correlating meteorological variables with historical PV data.

This paper proposes a novel GBDT-BiLSTM PV power prediction method. (1) Our model capitalizes on historical weather forecast data and PV generation records to decipher the underlying relationships between weather forecast data and power output. This method effectively rectifies the discrepancies between actual weather conditions and their forecasts in relation to PV generation, thus mitigating the inherent drawback of traditional NWP models that heavily depend on weather accuracy. (2) We harnessed gradient boosted Decision Trees (GBDT) to fit the training data through gradient enhancement. Moreover, the Bidirectional Long Short-Term Memory (BiLSTM), operating under the Adam algorithm, enhances the learning capacity of original PV timing information, thereby streamlining computation times. (3) Through the Teacher Forcing mechanism, we innovatively replace actual values with predictions from GBDT, facilitating corrections to the LSTM model. This, combined with gradient descent, adaptively learns the relationship between historical PV power outputs and meteorological variables. In comparisons with other algorithms in day-ahead power generation forecasting experiments, our method showcases enhanced prediction accuracy and generalization capability.

The remainder of the paper is organized as follows: “Developing forecasting model” presents the proposed GBDT and BiLSTM models. Case studies are presented in “Case studies and simulation results” to evaluate the performance of the proposed forecasting model. Conclusions are drawn in “Discussion”.

**Figure 1:**
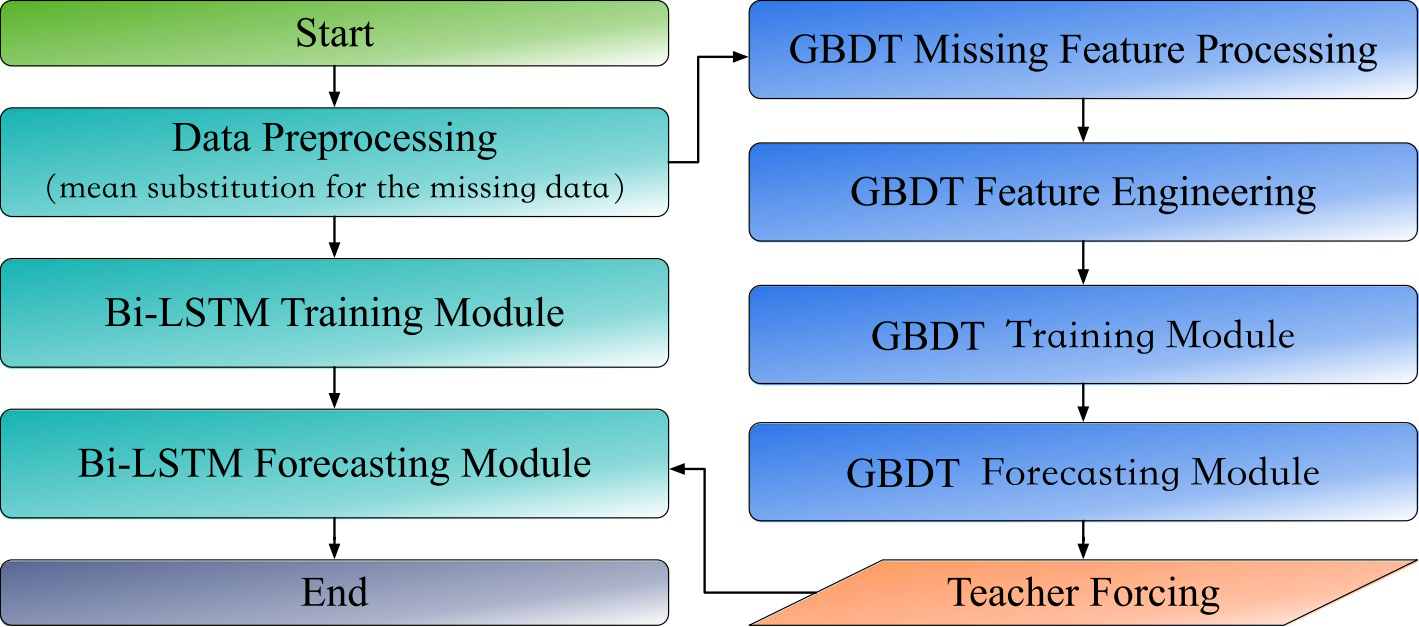
Day-ahead PV prediction via GBDT-BiLSTM hybrid model.

## Developing forecasting model

Traditional models often struggle with the complex interactions and temporal dependencies in weather data. Some might excel in capturing structural patterns but may not handle sequential information efficiently, and vice versa. To address these challenges, we introduce a hybrid model that synergizes the strengths of GBDT and BiLSTM. GBDT excels in modeling structural patterns, while BiLSTM is adept at processing time series data. This fusion ensures efficient structural pattern recognition and seamless sequential information processing, as illustrated in Fig. 1. The intricacies of this combined approach and its impact on PV forecasting accuracy are elaborated in the subsequent sections.

### GBDT

#### Gradient boosting

**Figure 2:**
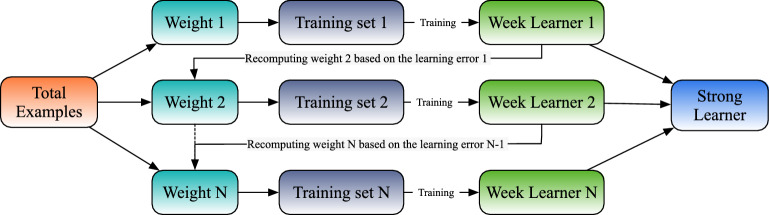
A principle schematic diagram of gradient boosting.

The gradient boosting (GB) is often used to build regression models^19^. It approaches the minimum loss value in the gradient direction through repetition and iteration. As Fig. 2 shows, the lost of original data is minimized in iterations, which produces a loss function for weak learners. After N times iterations, a more accurate and stronger learner can be trained. This algorithm can solve the optimization problem of spatial functions.

#### Decision tree

Decision tree is a supervised learning model based on the tree structure^20^. It utilizes the concept of entropy in informatics to solve basic classification and regression problems. The tree structure of the model can be adjusted in accordance with the characteristics of the data set^21^, making decision tree models flexible in handling various types of data, including continuous and discrete values.

In this paper, we use the least square regression tree to express the model, which adapts to the impurity function of the regression tree. The standard formula of the decision tree can be shown as:

1 $$\begin{aligned} F\left( X,\left\{ X_1, X_2\right\} \right) =\sum _{x_i \in X_1}\left( y_i-\hat{y}_1\right) ^2+\sum _{x_i \in X_2}\left( y_i-\hat{y}_2\right) ^2 \end{aligned}$$

#### GBDT

Based on the above algorithm, the GBDT algorithm can be viewed as a regression model that combines all the weak learners of the model into a strong learner. The specific algorithm is as follows:

Step 1: Initialize the weak learner, assuming a loss function L(y, f(x)). The mean value of c can be set as the mean value of sample y.

2 $$\begin{aligned} g_0(x)=\arg \min _c \sum _{i=1}^n L\left( y_i, c\right) \end{aligned}$$

Step 2: Make M times iterations. First estimate the residual.

3 $$\begin{aligned} r_{i m}=-\left[ \frac{\partial L\left( y_i, f\left( x_i\right) \right) }{\partial f\left( x_i\right) }\right] _{f(x)=f_{m-1}(x)} \quad i=1, \ldots , n \end{aligned}$$

Generate a regression tree from $\left\{ \left( x_i, r_{i m}\right) , i=1 \ldots n\right\}$, and the node area of the number m tree is $R_{m j}(j=1,2, \ldots , J)$. Then find the optimal $\mathscr {C}_{m j}$ for j=1,2,…,n, and minimize the loss function. The formula can be shown as:

4 $$\begin{aligned} c_{m j}= & {} \arg \min _c \sum x_i \in R_{m j} L\left( y_i, f_{m-1}\left( x_i\right) +c\right) \end{aligned}$$

5 $$\begin{aligned} h_m\left( x_i\right)= & {} \sum _{j=1}^{\left| R_m\right| } c_{m j} I\left( x_i \in R_{m j}\right) \end{aligned}$$

Step 3: Update the model:

6 $$\begin{aligned} g_m(x)=g_{m-1}(x)+\sum _{j=1}^J c_{m j} I\left( x_i \in R_{m j}\right) \end{aligned}$$

Step 4: Until the completion of N iterations, we can use an additive model to represent the prediction function G(x) with higher accuracy:

7 $$\begin{aligned} G(x)=\sum _{i=1}^M \sum _{j=1}^J c_{m j} I\left( x_i \in R_{m j}\right) \end{aligned}$$

We still assume that the training set has n samples, the specific model is as follows:
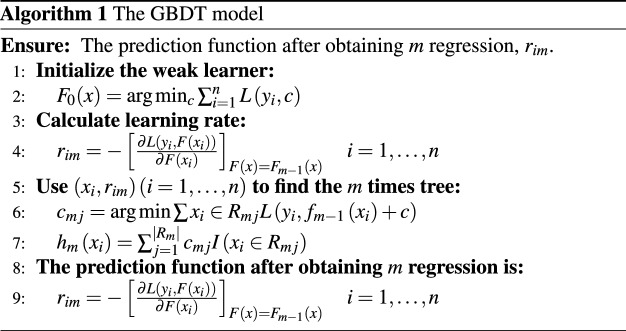


GBDT have been widely used for modeling various patterns and simulating the NWP model with weather information. However, GBDT lacks the ability to learn from sequential data, a critical factor in predicting time-dependent phenomena like weather patterns.

### LSTM

**Figure 3:**
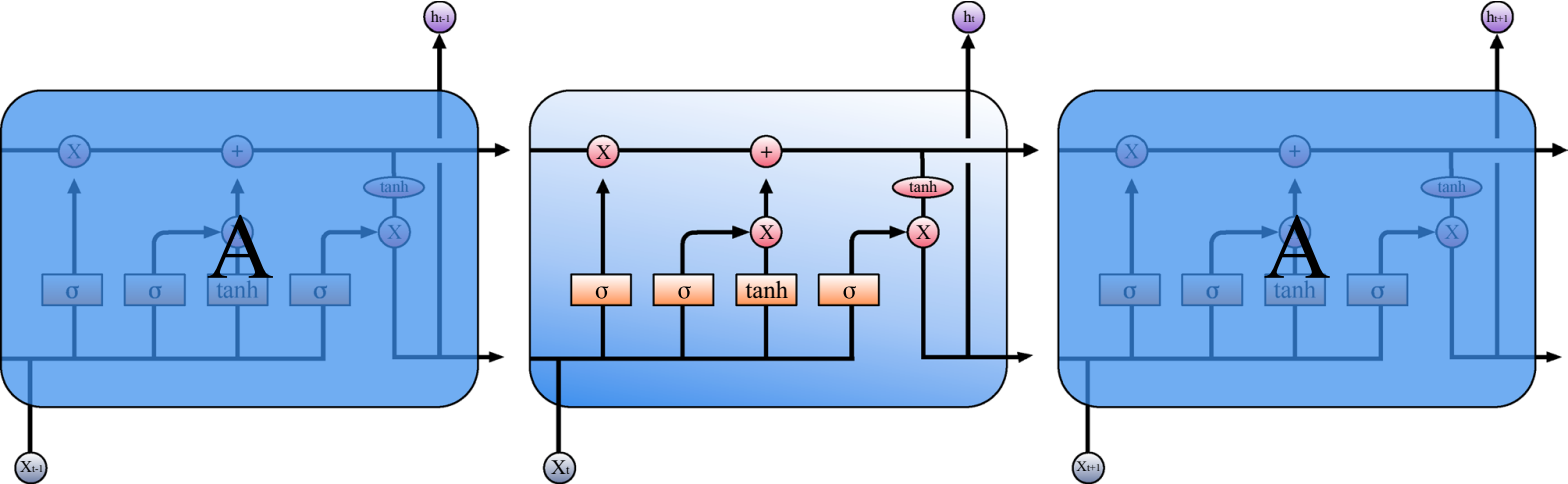
LSTM logic diagram. Where ht means hidden state, xt means input, $\sigma$ means sigmoid link layer, x means point wise multiplication, + means point wise addition.

To address this limitation, we introduce the use of Long Short-Term Memory, which is well-suited for processing time series data. LSTM is a special Recurrent Neural Network (RNN) model^22^ that utilizes the long-term dependence of valid data samples and has a long-term memory function at the same time. Compared with the ordinary RNN model, it has three more controllers: input gate, output gate and forget gate, which can effectively solve the problem of perception distance loss during conversion training in the RNN network^23^. The logic diagram is shown in Fig. 3. In addition, the special neuron mechanism of LSTM has the powerful information transmission and memory storage functions, so it is easier to capture the dependence of time series, reduces the information degradation rate, and increases the depth of calculation. Researchers nowadays take the advantage of the regularity of solar radiation to apply LSTM for short-term prediction, this algorithm is particularly effective when the data sample is limited. The following is a working logic of LSTM^24^.

The first step in LSTM is to decide what information we are going to throw away from the cell state. This decision is made by a sigmoid layer called the “forget gate layer”. The forget gate can be expressed as:

8 $$\begin{aligned} f_t=\sigma \left( W_f \cdot \left[ h_{t-1}, x_t\right] +b_f\right) \end{aligned}$$

h and x respectively represent two vectors associated with each other. $W_f$ is the weight coefficient matrix; $\sigma$ is the sigmoid function; $b_f$ is the bias parameter.

The second step is to decide what new information we are going to store in the two parts of the cell state. First, “input gate layer”, a sigmoid layer, decides which values we will update. Next, a tanh layer creates a vector of new candidate values, $\tilde{C}_t$, that could be added to the state.

9 $$\begin{aligned} \begin{aligned} i_t&=\sigma \left( W_i \cdot \left[ h_{t-1}, x_t\right] +b_i\right) \\ \tilde{C}_t&=\tanh \left( W_c \cdot \left[ h_{t-1}, x_t\right] +b_c\right) \end{aligned} \end{aligned}$$

tanh is the tanh function; the weight coefficient matrix of the first part of $\textrm{W}_i$; $\textrm{W}_c$ is the weight coefficient matrix of the second part; $b_i$ and $b_c$ are used as the bias item parameters of this input level.

The third step is to update the old cell state $C_{t-1}$ into the new cell state $C_t$. Then we add $i_t * \tilde{C}_t$.

10 $$\begin{aligned} C_t=f_t * C_{t-1}+i_t * \tilde{C}_t \end{aligned}$$

Following that the numerical unit coefficient matrix of the state at the moment is multiplied by the tanh function to get the final output result.

11 $$\begin{aligned} \begin{aligned} o_t&=\sigma \left( W_o\left[ h_{t-1}, x_t\right] +b_o\right) \\ h_t&=o_t * \tanh \left( C_t\right) \end{aligned} \end{aligned}$$

In the above formula, $W_o$ represents the weight coefficient matrix of the output level; and $b_o$ represents the bias coefficient of the output level.

#### BiLSTM

While LSTM offers significant advantages in handling sequential information, there exists an opportunity to further enhance the learning process. This leads us to explore Bi-directional Long Short-Term Memory (BiLSTM), a variant of LSTM.

**Figure 4:**
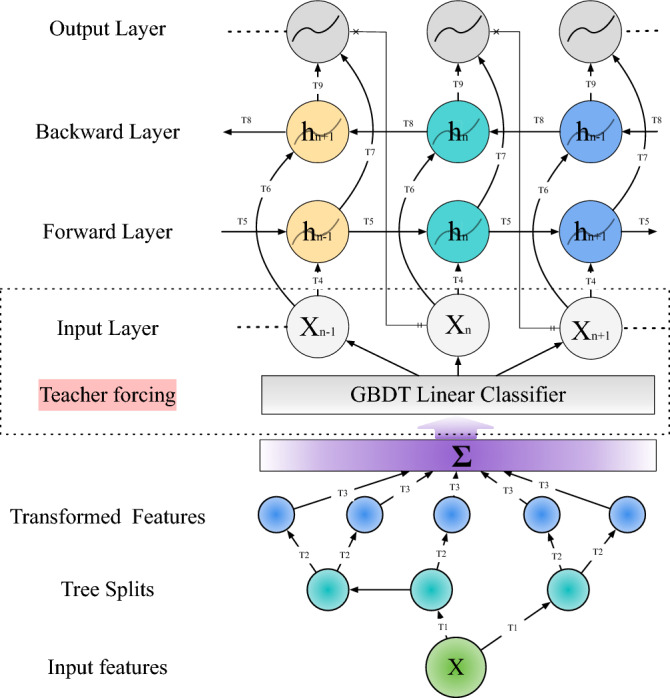
GBDT-BiLSTM logic diagram. Where ht means hidden state, Xn means LSTM input, x means GBDT weather data. This picture shows the overall logic of the GBDT-BiLSTM model. The output F(X) of GBDT is used as the input value of LSTM through the Teacher forcing mechanism.

BiLSTM is composed of forward LSTM and backward LSTM^25^. Just as Fig. 4 shows, one string goes from the front to the back and the other string does the opposite. In Fig. 4, we can see that the input of LSTM passes through Backward Layer and Forward Layer from T4 and T6 directions, respectively. Finally, the output value is obtained after splicing T7 and T9. These two strings of LSTM results are calculated to obtain a composite result, which can better capture the time relationship for PV prediction^26^.

#### Adam algorithm in BiLSTM

The Adam (adaptive moment estimation) algorithm is a gradient descent algorithm for optimizing neural network models. Combining the ideas of gradient descent and momentum optimization, it converges on the global optimal solution faster and is relatively robust to the selection of hyperparameters.

The Adam algorithm computes its mean and an exponentially weighted moving average of the mean of squared gradients, respectively. These moving averages are calculated by the following formulas:

12 $$\begin{aligned} m=\beta _1 * m+\left( 1-\beta _1\right) * g\\ v=\beta _2 * v+\left( 1-\beta _2\right) * g^2 \end{aligned}$$

Among them, m represents the average gradient, v represents the average square gradient, $\beta _1$ and $\beta _2$ are two hyperparameters, usually set to 0.9 and 0.999, respectively.

Calculate the bias correction coefficient: since the initial values of the exponentially weighted moving averages are all 0, they need to be bias corrected to avoid the problem that the update step size is too large or too small in the early stage of training. These bias correction factors are calculated by the following formulas, respectively:

13 $$\begin{aligned} m_{\text {hat }}=m /\left( 1-\beta _1^t\right) \\v_{\text {hat }}=v /\left( 1-\beta _2^t\right) \end{aligned}$$

Among them, t represents the number of current iterations. Since t will increase by 1 in each iteration, the power of $\beta _1$ and $\beta _2$ will continue to increase. Calculating the update step size: based on estimates of the average gradient and the mean of the squared gradients, the update step size is calculated by the following formula:

14 $$\begin{aligned} \Delta _\theta =-\alpha * m_{\text {hat }} / \sqrt{\nu _{\text {hat }}}+\varepsilon \end{aligned}$$

Among them, $\alpha$ is the learning rate, which controls the size of the update step, and epsilon is a small number to prevent the denominator from being 0. The sign of the update step is a negative sign, because the Adam algorithm is a gradient descent algorithm, and the parameters need to be updated along the opposite direction of the gradient.

### GBDT-LSTM model with teacher forcing

Having delineated the individual merits of GBDT and BiLSTM in previous sections, we now turn our attention to their fusion. The integration of GBDT and BiLSTM aims to leverage the unique strengths of both methods, but it poses a challenge in the context of day-ahead prediction of PV power generation.

As being detailed in the LSTM section, the RNN prediction ability in the early stage of its training iteration process is often weak, leading to ineffective generation results. This can cascade through the learning of subsequent units, causing a compounding effect. To tackle this, we introduce the Teacher Forcing mechanism, a rapid and efficient method to train the cyclic neural network model. Rather than using the output of the previous state as input for the next state, Teacher Forcing updates it with the newly available ground truth data.

However, in the domain of day-ahead photovoltaic prediction, power plants are required to submit their estimated 24-h power generation data to the market trading organization in advance. The teacher forcing mechanism, when used to constrain the LSTM model, can only be updated hourly, making it unsuitable to predict the 24-hourly power generation of the next day. This limitation underscores the need for an innovative approach to address the time scale problem.

To address this, our model utilizes the prediction results of GBDT to replace the conventional Ground Truth Predict data. This novel method not only improves the accuracy of the LSTM model but also effectively solves the aforementioned time scale problem.

The proposed GBDT-LSTM model operates in a sequence of steps to accurately predict PV power generation. The algorithm’s computational flowchart can be referred to in Fig. 4. Here, we present the step-by-step process of the model:

1. Input features formation: The weather data and historical PV data are combined to form the input features $X$, serving as the initial data for the model.
2. Tree splits and features transformation: The input features $X$ are processed through tree splits and features transformation. This step refines the data and makes it suitable for the GBDT model.
3. GBDT model prediction: An updated learner $G(x)$ is derived from the transformed features, resulting in the GBDT model’s prediction of PV power output. $G(x)$ represents the output of the GBDT model and contains valuable information about the underlying structural patterns of the data.
4. Integration with BiLSTM using TF mechanism: Utilizing the TF mechanism, the results of $G(x)$ are fed into the BiLSTM’s $X_{(n)}$. This step ensures that the time-dependent nature of the data is captured, and the information from the GBDT model is integrated with the sequential processing capability of the LSTM model.
5. BiLSTM neural units computation: The data then passes through the T1 to T9 neural units of the BiLSTM model. During this stage, computations are performed in both the Backward Hidden Layer and Forward Hidden Layer to calculate the two layers of $h_n$. This two-layer structure enhances the model’s ability to analyze the sequential information in the data.
6. Output layer fusion: Finally, the fused model prediction result is obtained at the output layer. This result is a synthesis of the structural insights provided by the GBDT model and the time series processing power of the LSTM model, leading to a more robust and accurate prediction of PV power generation.

The synergy between GBDT and BiLSTM, facilitated by the TF mechanism, creates a powerful model capable of handling both the structural and temporal aspects of PV prediction. The above steps collectively contribute to a comprehensive solution tailored for the specific challenges of PV power generation forecasting. And the mathematical output result of this model can be defined as:

15 $$\begin{aligned} \psi _t=O_t * \tanh \left( C_t\right) \end{aligned}$$

where $O_t$ uses a sigmiod function to represent the output content, which can be defined as:

16 $$\begin{aligned} O_t=\sigma \left( W_0\left[ h_{t-1}, G(x)\right] +b_0\right) \end{aligned}$$

At the same time, we can redefine the update layer and forget layer functions in a LSTM cell as:

17 $$\begin{aligned} \tilde{c}_t= & {} \tanh \left( w_c \cdot \left[ h_{t-1}, G(x)\right] +b_c\right) \nonumber \\ i_t= & {} \sigma \left( w_i-\left[ h_{t-1}, G(x)\right] +b_c\right) \end{aligned}$$

18 $$\begin{aligned} z_t= & {} \sigma \left( W_f \cdot \left[ h_{t-1}, G(x)\right] +b_f\right) \end{aligned}$$

$G(x)$ represents the output result of the GBDT PV prediction portion, where $x$ consists of the input features formed from historical weather data and historical photovoltaic (PV) series. This output captures the structural patterns within the data, specifically the PV prediction result generated by GBDT based on the structural relationship between weather conditions and historical PV series. $\psi _t$ symbolizes the final output result of the model, synthesizing the structural insights from GBDT with the time-series processing capability of LSTM, to provide a more comprehensive and accurate forecast.

## Case studies and simulation results

To validate the efficacy of the proposed algorithm, various tests have been carried out based on the Pytorch operating environment using Azure Linux 23.02, 06 system with the virtual machine size being Standard-NC6 (6 cores, 56 GB RAM, 380 GB disk) and the processing unit is GPU-1 $\times$ NVIDIA Tesla K80.

The experimental data used in this study was collected from a PV power station in Ganzi Tibetan Autonomous Prefecture, Sichuan Province, China. The data span a period of 18 months, from the second half of 2020 to 2021. This includes meteorological forecast data from the European Central Weather Center and power generation data from the PV power station. The meteorological data, which encompass temperature (2 m above the ground), humidity (2 m above the ground), air pressure, precipitation, ground surface wind speed (10 m above the ground), wind direction (10 m above the ground), and total radiation (from the PV panels of the power station), were collected at 1-h intervals. However, the power generation data, derived from the historical records of the PV power station, were collected at daily intervals. In addition, before the data preprocessing phase, we utilized the Radial Basis Function (RBF) interpolation to align both the hourly weather forecast data and the daily data from the PV power station, which includes temperature, irradiance, wind speed, and humidity. This ensured consistency and seamless integration for our predictive model.

### Data preprocessing

**Figure 5:**
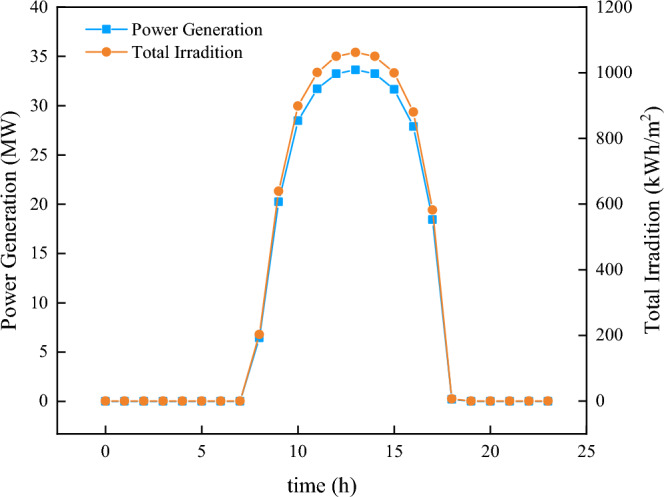
The comparison between power generation and the PV irradiation.

In this experiment, the last month data were used as the validation set, with an hourly sampling rate. The time window sampling used was a 24-h period for both the GBDT model and the LSTM model. And the experiment focuses on the daily change in solar radiation. The total PV power generation for a day was discretized into hourly increments, with the discretized function representing the daily change in solar radiation. Figure 5 compares the 24-h change in irradiation with the discrete change in power generation.

**Figure 6:**
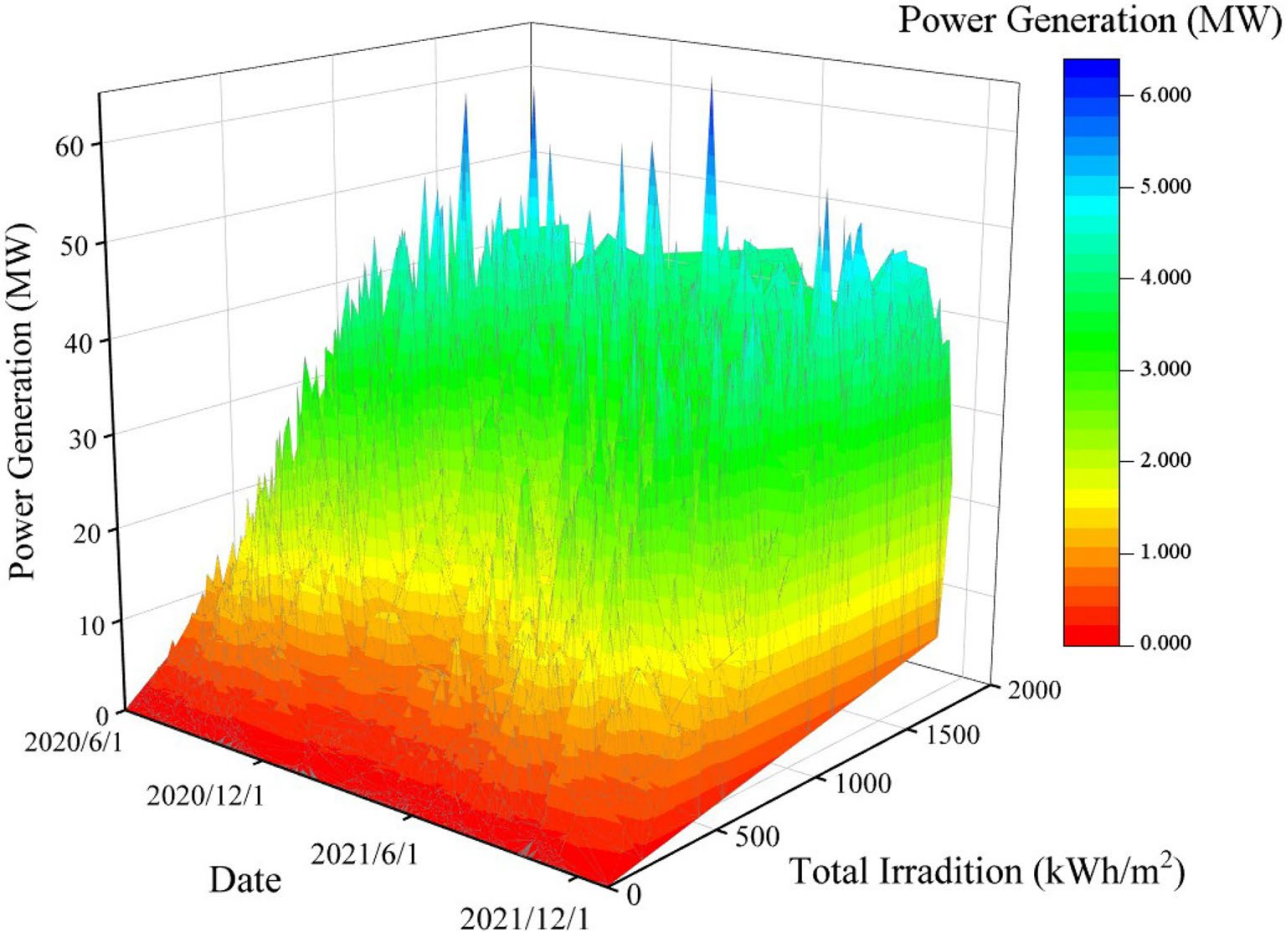
Three-dimensional data map of the total radiant power generation from June 2020 to December 2021.

The outlier data are removed and their places as well as missing data are filled in by Difference Filling methods in order to reduce the impacts of the bad data on the LSTM algorithm. The PV power generation data for 18 months are displayed in Fig. 6.

### GBDT modeling results

**Figure 7:**
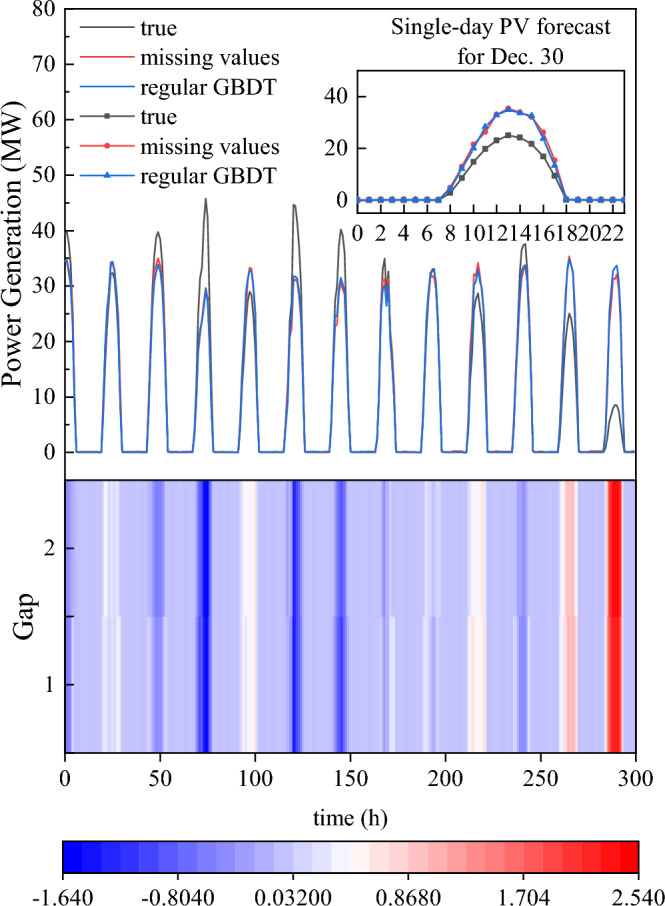
Last 300 h of the GBDT model test set.

Based on the NWP physical model, this experiment included the weather data, such as temperature, moderate humidity, precipitation, and wind speed. However, the NWP model requires high accuracy and data breadth in actual engineering applications. To address this, we adjusted the weight of missing values, and allowed prediction of future power generation even in the absence of certain weather features. To demonstrate this, we randomly set 30% of the data for each feature, excluding solar radiation, to missing values (− 1). And then we refitted the training data and completed the prediction. The simulation fitting curve for the last 300 h of the GBDT algorithm test set is shown in Fig. 7.

Figure 7 result shows that the prediction accuracy has declined after the operation in Missing value. However, our algorithm successfully capture the strong correlations between solar radiation characteristics and PV power generation, thus the absence of weather measurements to 30% has little impact on the prediction results and the prediction error is controlled within a reasonable range. Similarly, the 30% missing values set in the experiment have also been adjusted and modified. After the data verification, the root mean square error of this model was 2.0162 (when all features were intact) and 2.150 (when some features were missing), which verifies the robustness of the GBDT model in this part.

### GBDT-BiLSTM modeling results

**Figure 8:**
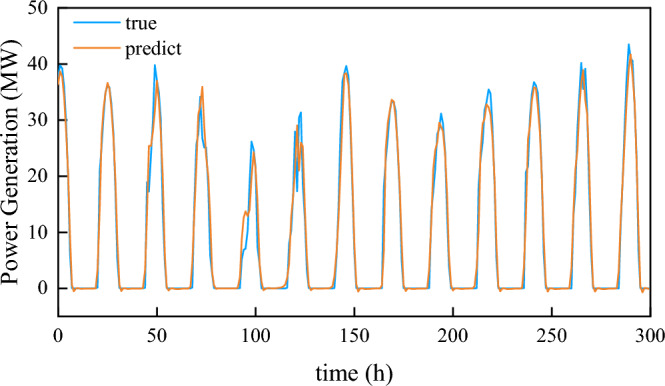
Last 300 h of the BiLSTM model training set. The mse and mae of the training set are 0.054821357 and 0.12106604, respectively. Compared with 0.17525618664000464 and 0.2018899794652092 of the GBDT model, it can be seen clearly that the fitting effect of the BiLSTM model is better than that of the GBDT model.

In this phase of experiment, we used the deep learning algorithm of the BiLSTM model to simulate and predict historical power generation data first. Different from the conventional BiLSTM model, we used the prediction result of GBDT to replace the ground truth requirements in Teacher Forcing mechanism when updating the LSTM model. BiLSTM had the same time partition criterion with the GBDT model. The environment of BiLSTM’s batch size was set to 1024. The Adam optimization algorithm is used here with the mean square error as the loss function and 200 times as data traversals. The training result is shown in Fig. 8

**Figure 9:**
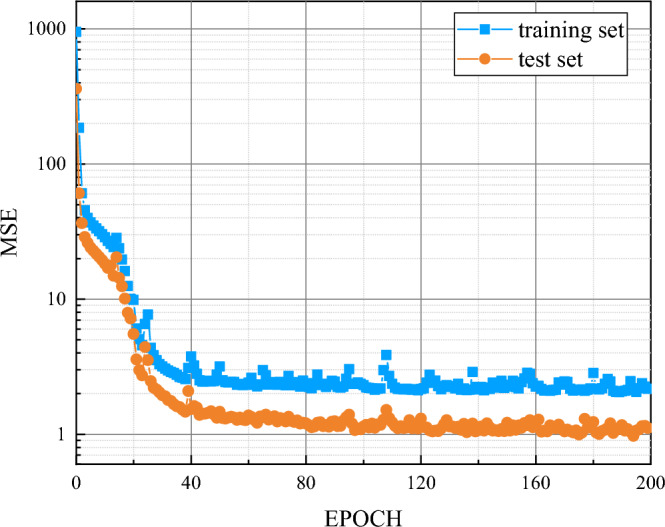
MSE curves of the model training set and the test set.

Secondly, we used the test and data to perform the mse fitting test and save the mse change curve. After 200 iterations, the mse curves of the model training set and the test set tended to be consistent. The fitting results are shown in Fig. 9.

**Figure 10:**
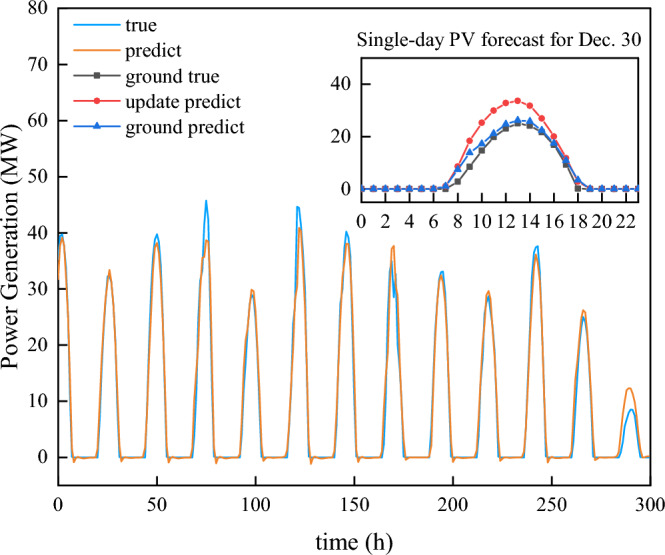
Last 300 h of the BiLSTM model test set.

Then we output the last 300 h training curve of the test set. Compared with Fig. 10, it can be seen that the fitting effect of the BiLSTM model in the day-ahead PV prediction model is better.

**Figure 11:**
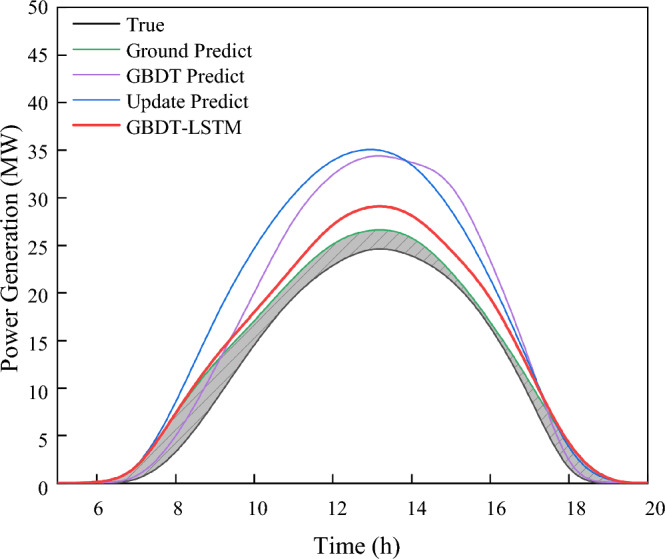
GBDT-BiLSTM predicts power generation on December 30. This picture includes five models: LSTM, GBDT, update predict LSTM, GBDT-LSTM and True.

Third, we imported the Teacher Forcing mechanism and used the prediction of GBDT as the ground truth data to update the prediction result of BiLSTM. Temperature, air humidity, air pressure, precipitation, surface wind speed and total radiation were imported as features and the time scale remained unchanged. The final prediction result is shown in Fig. 11.

Finally, as shown in Fig. 11, there are five curves in this picture, among which the black one is the ground truth. The green one is the predicted value based on the Teacher Forcing mechanism provided by true power generation data. The red one is the GBDT-BiLSTM model, which is closer to the true value than the predictions of GBDT and the single BiLSTM models. The shaded region between the ground truth and the actual values represents the difference between them, signifying the ideal performance boundary of the model and indicating its potential upper limit.

### Model comparison

**Figure 12:**
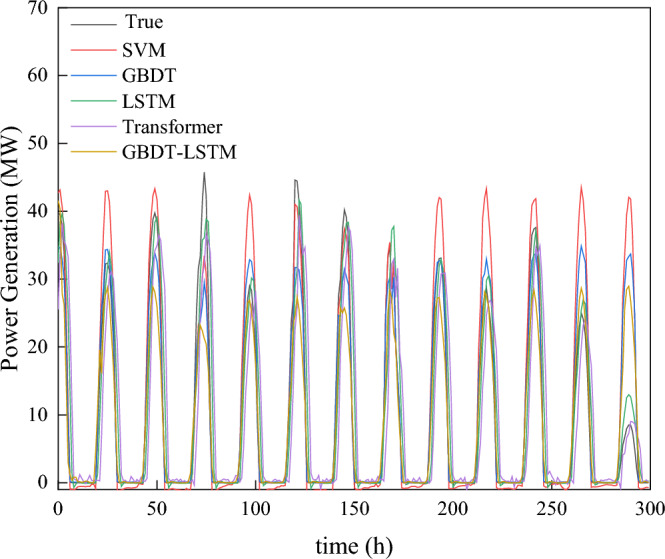
Last 300-h forecast curves of multiple model test sets. This picture includes five models: SVM, GBDT, update predict LSTM, GBDT-LSTM and update predict transformer.

In order to verify the effectiveness of the new model, this paper compares the new model with SVM^27^, Transformer^28^, GBDT and LSTM models using the same database. The comparison results are shown in Fig. 12. In the figure presented, it is evident that the prediction fit of the newly proposed model in the last 300 h of the test set is the closest to the actual value.

**Figure 13:**
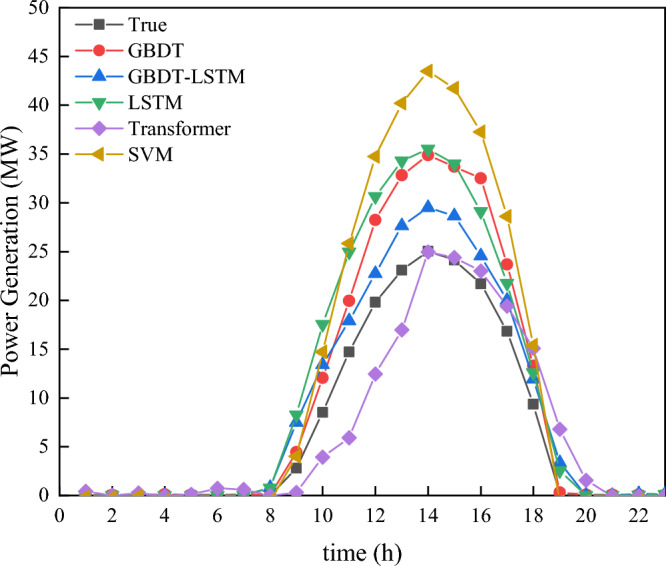
Multiple models predict power generation on December 30. This picture includes SVM, GBDT, update predict LSTM, GBDT-LSTM and ground true predict transformer.

In Fig. 13, December 30th serves as a case study to demonstrate the comparison between the new model and other models. Despite the time scale problem associated with the teacher forcing method in the domain of day-ahead photovoltaic prediction, the use of GBDT prediction results as a replacement for conventional Ground Truth Predict data has not only enhanced the accuracy but also solved this unique challenge. Furthermore, our results underscore that our model exhibits robust performance, even when confronted with missing or inaccurate weather data.

Focusing on the test set error values, the BiLSTM model with teacher forcing achieves the lowest values in MAE (0.08), MSE (0.151), and MASE (0.05), indicating its superior prediction accuracy. The presence of LSTM with teacher forcing in the final model comparison results provides a pertinent benchmark, demonstrating the innovative approach of the hybrid GBDT-BiLSTM model. In addition, the GBDT-BiLSTM hybrid model also demonstrates good performance with an MAE of 0.112, MSE of 0.141, and MASE of 0.102, exhibiting an enhanced capability that approaches the ideal case of teacher forcing. On the other hand, other models such as SVM, GBDT, Improved GBDT, and Transformer exhibit relatively poorer performance across all metrics.

In the comparison of Fig. 13 and Table 1, our model stands out in terms of both prediction accuracy and robustness against inconsistent weather data. The model is especially effective in situations where the weather data might be incomplete or not entirely accurate. This makes our model a reliable choice for real-world applications, ensuring consistent prediction even when faced with challenges in data quality.

**Table 1:** Comparison of prediction performance.

Prediction model	Training set		Test set	
	mae	mse	mase	mse
TF-LSTM (ideal)	0.08	0.151	0.05	0.124
SVM	0.253	0.216	0.219	0.187
GBDT	0.191	0.141	0.179	0.197
Improved GBDT	0.199	0.150	0.203	0.189
Transformer	0.339	0.312	0.345	0.297
GBDT-BiLSTM	0.112	0.141	0.102	0.128

## Discussion

This paper proposes a new algorithm combining GBDT and bidirectional LSTM to predict the power generation output of PV power plants, which improves the prediction accuracy of day-ahead PV model. The conclusions are as follows:

1. With NWP method to calculate 7 weather characteristics affected PV power generation, the improved GBDT model increases the prediction accuracy of PV power effectively, and adjusts the missing rate of random samples. Even under the condition of missing value, the model can also output the satisfactory predicted values and maintained the prediction accuracy, which reduce the model reliance on the accuracy of the weather data.
2. Since PV power generation is strongly correlated with solar radiation and changes regularly over time, this paper uses the Bidirectional Long-Short Term Memory model for PV power generation prediction, which can improve the prediction effect of the model under weak light condition. This model uses the Adam optimization algorithm to speed up the optimization process and utilizes the adaptive learning rate of gradient descent to improve the learning ability. It can also improve the fitting effect with the GBDT model and the long-term prediction accuracy.
3. By combining the Teacher Forcing mechanism with BiLSTM and the improved GBDT model, this model reduces the overfitting problem. The Teacher forcing mechanism, typically employing real power generation values, provides an effective method to calibrate the BiLSTM model. However, as it updates only hourly, this method is unsuitable for predicting the 24-h power generation required for our task. Since using the actual real-time values for Teacher Forcing is impractical in our context, we instead utilize GBDT predictions. This innovative adaptation aligns more appropriately with the time-scale demands of our domain, without sacrificing the benefits of the Teacher Forcing technique.
4. Finally, compared to other methods, the fused GBDT-BiLSTM model improved the accuracy of day-ahead PV forecasting due to the consideration of time series and various weather characteristics. Comparing it with the LSTM model that utilizes Teacher Forcing provides a more comprehensive demonstration of the superiority of the fusion model, especially in the context of applying Teacher Forcing in our specific scenario.

The numerical experiments suggest that the GBDT-BiLSTM hybrid model offers enhanced accuracy and practicality compared to some existing models. Its improved prediction capabilities and ability to manage time-scale intricacies represent a notable contribution to PV forecasting. Beyond its immediate application in solar power generation, it paves the way for further exploration in machine and deep learning predictive models across various sectors. Importantly, the model seeks to better align how photovoltaic stations communicate with the grid, aiming for closer correspondence between predicted and actual outputs. This alignment could potentially offer economic advantages by lessening the need for backup power and reducing grid-related financial implications. Furthermore, this model’s precision may assist grid operators in making informed decisions, aiming for a more stable power grid and subsequently supporting industrial and societal stability (Supplementary Information).

### Supplementary Information

Supplementary Information.

## Data Availability

The datasets used and/or analysed during the current study available from the corresponding author on reasonable request.
